# Piloting electronic informed consenting in a pneumococcal human infection study in Blantyre, Malawi

**DOI:** 10.12688/wellcomeopenres.20770.1

**Published:** 2024-04-29

**Authors:** Clara Ngoliwa, Chikondi Chakwiya, Joel Gondwe, Edna Nsomba, Vitumbiko Nkhoma, Modesta Reuben, Linda Chantunga, Pemphero Liwonde, Edward Mangani, Evaristar Kudowa, Lumbani Makhaza, Neema Toto, Tiferanji Sochera, Tarsizio Chikaonda, Ben Morton, Marc Y.R. Henrion, Dingase Dula, Stephen B. Gordon, Anthony E. Chirwa

**Affiliations:** 1 Malawi-Liverpool-Wellcome Trust Clinical Research Programme, Blantyre, Malawi; 2 Department of Medicine, Queen Elizabeth Central Hospital, Blantyre, Malawi; 3 Department of Clinical Sciences, Liverpool School of Tropical Medicine, Liverpool, UK; 4 Department of Clinical Sciences, Liverpool University Hospitals NHS Foundation Trust, Liverpool, UK

**Keywords:** Electronic informed consent, Open Data Kit, clinical trial, Streptococcus pneumoniae, randomized control trial.

## Abstract

**Background:**

Electronic consent can potentially improve accuracy, workflow, and overall patient experience in clinical research but has not been used in Malawi, owing to uncertainty about data security and technical support.

**Objectives:**

We explored the feasibility of using electronic consent (e-consent) in an ongoing human infection study in Blantyre Malawi. We dual-consented participants by both electronic and paper methods to assess the feasibility of electronic consent, and then compared benefits and challenges of the two methods.

**Methods:**

The approved paper consent forms were digitized using Open Data Kit (ODK). Following participant information giving by the research staff, healthy literate adult participants with no audio-visual impairments completed a self-administered e-consent and provided an electronic signature. Signed e-consent forms were uploaded to a secure study server. While the participants were in clinic, the signed electronic consent form was printed as a copy for the participant. The feasibility, advantages and disadvantages including data safety consideration for e-consenting were evaluated by exploring issues surrounding use of e-consenting versus paper-based consenting. Consent forms were analysed by research staff for errors such as overwriting and legibility.

**Results:**

We piloted 109 participants to e-consenting. It was found to be user friendly, had 0% (n 0/109) errors compared to 43.1% (n 47/109) in paper based methods along with enhanced data safety. The challenges included difficult digitization of ethics stamped documents, volunteer unfamiliarity with tablet user interface and its requirement for a working internet and printer.

**Conclusion:**

E-consenting was feasible but required additional resource investment. Benefits included error minimization and data security.

## Plain Language Summary

Informed consent involves educating a patient or participant about the risks, benefits and alternatives of a procedure or clinical research in a format and language that they can understand to achieve voluntary participation. Traditionally paper-based consent has been used but it is not without its limitations thus the need to introduce electronic consent. Electronic consent involves the use of electronic devices to deliver a variety of media including video as well as written words to convey the study details and then secure digital recording to save the informed consent.

We piloted electronic consent in an ongoing human infection study to assess feasibility in Malawi. The approved paper consent forms were digitized and uploaded to an electronic platform. Participants completed dual consent of paper and electronic consent in that order. We then compared issues arising from using both methods.

We found that e-consent was feasible. It proved to be reliable, and minimized documentation errors. We noted, however, that electronic consent could not be done in technologically challenged settings as it required internet connection to help upload forms to secure servers. In addition, researchers are still required to provide printed proof of consent and so current practice would also require a printer to be available. When infrastructure limitations are overcome, e-consent offers improved participant experience and increased data reliability.

## Introduction

Informed consent is an integral feature of the ethical conduct of a trial, it is a key step before medical procedures and research participation. It involves educating a patient or participant about the risks, benefits and alternatives of a procedure or clinical research in a format and language that is comprehensible, and getting their voluntary participation ^1^.

Paper-based patient information sheets followed by obtaining a handwritten signature is the default method used in hospitals and research institutions. However, this method is not without limitations, that include time and effort demand, documentation errors (inconsistencies in clarity, completeness, legibility), and need for physical storage space ^2^.

Electronic informed consent (e-consent or digital consent) has been introduced to supplement or replace paper-based consenting in many settings. This method uses electronic devices such as cellphones, tablets or IPads with installed software that enables delivery of information to the potential volunteer, who demonstrate consent by providing an electronic signature or thumbprint. An electronic Signature (e-Signature) is an electronic sound, symbol, or process, attached to or logically associated with an electronic record and used by a person with the intent to sign a record ^3^. E-consent can be either in-person; where the person obtaining consent and potential volunteer are in the same physical location, or remote, where the potential participant reviews the consent document in the physical absence of the person obtaining consent. In the latter, the consent process may utilize web-based software, email, postal service, mail, or a mobile phone to convey and retrieve signed research documents ^4^.

Advantages of electronic informed consent include flexibility to allow remote consenting for the volunteer or their legally authorized representative, time-saving, when surveys are completed before in-person visits and immediate consultation with family and friends ^5^. Additional advantages are user-friendliness, less workload for staff, more privacy and control for volunteers, better comprehension particularly when audio-visual enhancements are used ^6^. However e-consenting is not without fault, as a tablet-based informed consent in general, has the potential limitation of the expense of developing the initial infrastructure and technology to manage and validate online documents. Additionally, there may be challenges regarding the need for a stable secure Wi-Fi network or cellular data coverage to allow for data delivery and updates to electronic consents ^7^.

## Methods

### Ethics

A proposal to pilot e-consenting and to consider introduction of the method to ongoing and future studies at the Malawi-Liverpool-Wellcome Programme (MLW) was presented to the National Health Science Research Committee (NHSRC) at a symposium in October 2021 by the Data Management Support Unit and Clinical Research Support Unit leads at MLW. NHSRC approved the proposal on 29 October 2021. Pilot began on 8 ^th^ February 2022 and was completed on 9 ^th^ May 2022.

### Study design

The study was a cross sectional observational study. It was a pilot of electronic and paper-based informed consent to allow comparing the two consent processes in an ongoing clinical trial ^8, 9^. The clinical trial was registered with the Pan African Clinical Trials Registry (REF: PACTR202008503507113) on 03 August 2020 and conducted at the Malawi Liverpool Wellcome Programme (MLW) in Blantyre, Malawi between April 2021 and September 2022. Source populations were college students and community members within Blantyre.

### Recruitment to the study of consenting methods

Healthy, literate, adults aged 18 to 40 years with no visual or hearing impairments attending the research clinic to enrol in a human infection study of experimental human pneumococcal carriage were asked to participate. The study site was ward 3A Research Clinic at Queen Elizabeth Central Hospital. We sought verbal consent from participants to undergo dual consenting (paper-based and electronic consenting in that order). Consenting was in person and was done on screening visit.

### Procedure for comparing consent methods in the Human Infection Study (HIS)

Participants first completed a paper-based consent process to the HIS. Paper-based consent process was done face to face on the screening visit. Study staff using the participant information sheet first explained more about the study allowing for participants to ask questions regarding the study. After the discussion, participants agreeing to participate in the study were quizzed with 10 questions to assess understanding with 80% being the passing score. A score below 80% meant that the participant had not fully understood and so study staff had to repeat some of the information. Participants then went through the consent form adding their initials at the end of each statement to show "comprehension" and "agreement", after which both the participant and study staff signed the consent form respectively. This step was then repeated as one form had to be given to the participant. The remaining form was then filed in the investigator file and stored in a locked cabinet. 

Following completion of paper-based consenting, electronic consenting process was initiated. Study personnel first linked the consent form to a study assigned participant identification (PID) by scanning the barcodes. The form would then prompt the study personnel to write participant name and their name exactly as written on the paper-based consent form. Participants then self-administered e-consenting in the presence of study personnel for troubleshooting as not all participants were familiar with the tablet user interface. Participants were required to simply select I AGREE with a single tap. Participants then signed using a stylus pen or fingertip and study personnel countersigned at the end of the consenting process. The consent form was then exported to the data server using an internet connection, printed and a copy was provided to the participant.

At the end of the study the consent forms were assessed by the study nurses and doctors for errors related to eligibility of consent forms and completion of forms by both participants and study personnel. One was prohibited from analyzing a form they themselves had completed.

### Setting up e-consenting in ODK

Open Data Kit (ODK) is an open-source suite of tools designed to help users build information services ^10^. These tools provide the ability to design forms (build), collect data on mobile devices (collect), and organize data into a persistent store where it can be analysed (aggregate) ^11^. ODK allows users to fill out research forms offline and then send it to secure data servers using internet connection, which then allows easy access, viewing and downloading of the data. Electronic forms, identical to existing paper-based forms were designed using XLSForm syntax which is an intuitive language. This involved defining the questions or consent statement, response options and other relevant information like signature fields. Once the form was designed, it was converted into XML format using the XLSForm converter tool. The XML files were then uploaded to the ODK server or directly to a mobile device using the mobile app for data collection called ODK collect. Following approval from NHSRC, an identical copy of the NHSRC ethical approval stamp was affixed to the electronic CRF in ODK. Clinic staff were trained to navigate the ODK platform.

## Results

A total of 109 HIS participants were piloted for e-consenting (see Table 1). All 109 participants were consented using both paper-based and electronic consenting. 67.0% (n 73) of our volunteers were male and 33.0% (n 36) were female. E-consenting proved to be feasible; it was reliable as it reduced the error rate to 0% (n 0/109) compared to 43.1% (n 47/109), it was also an effective and convenient tool though it proved to be impossible to do when internet was unavailable (see Table 2). More than half of the participants in this study were college students 63.6% (n 70) and as a result had experience of using computers and tablet devices.

**Table 1.** Demographic characteristics of e-consent pilot study participants.

	Male	Female	Total
**n (%)**	73 (67.0%)	36 (33.0%)	109 (100%)
**Age (mean)**	27	27.8	27.3
**Previous computer experience n(%)**	63 (57.3%)	23 (20.9%)	86 (78.2%)
**Education level (Primary) n(%)**	6 (5.5%)	10 (9.1%)	16 (14.5)
**Secondary n(%)**	17 (15.5%)	7 (6.4%)	24 (21.8%)
**Tertiary n(%)**	50 (46.0%)	19 (17.3%)	69 (63.3%)

**Table 2.** Pros and cons of electronic consenting.

Category	Pros	Cons
**Staff ** **operations**	- Reduced documentation errors - Easy to access consent forms	There was need for dual consenting (paper and electronic) as a hybrid method was adopted
**Internet ** **facility**	- Enhanced data safety as documents were immediately saved to secure server thus preventing them from loss due to misfiling or misplacement especially if there was more than one study happening at a site	- Difficult to upload ethics stamped documents - Could not be done if internet was not available
**Storage ** **facility**	- Reduced the burden of filing as all forms are stored on data server	- Difficult to access data when the internet is unavailable

A pro/con discussion of e-consent and paper consenting methods was conducted between the authors and study staff and the results summarized in Table 2. 

### Documentation errors analysis

Of the 109 consent forms, 47 of the paper consent forms contained errors. Some forms contained more than one error. Many of the errors 29.4% (n 32) were due to writing mistakes (overwriting, corrections for wrong date, spellings etc.) from both study personnel and participants, and 13.8% (n 15) were due to missing information (e.g. participant identification) on some pages of the consent documents.

## Discussion

This study proved that e-consenting is feasible in our context. Dual consenting was administered to participants by nurses and doctors. E-consenting showed a 0% error rate as compared to 43.1% in paper-based consenting. In this study, we found that e-consenting is feasible if done in a technologically competent setting. The use of electronic consent was noted to have improved consent form accessibility and reduced the burden of the need to print all consent and information documents. It also helped in promoting data safety and reduced the risk of data breach.

### Previous studies

Paper-based consenting has been reported to have deficiencies associated with documentation errors ^12, 13^ hence the need for e-consenting. One study showed that e-consenting had a low error rate of 0.32% as compared to 7% of paper based consent ^14^. It also showed improved workflow for staff and high overall satisfaction ^14^. Another study showed that electronic consenting improved participant usability and satisfaction as compared to paper based consenting ^15^.

### Study strengths and limitations

The study included participants with different educational levels and different age groups. All forms used in e-consenting in this study were of ICH GCP standard and had undergone ethics committee approvals. Study staff had adequate training and were conversant with the ODK data collection tool.

This study was limited in that it was observational and our results are based on an analysis of one study. Lack of randomization of the two consenting processes is also another limitation. The study did not explore the inclusion of individuals with audio-visual impairments and illiterate participants as the main trial under which the study was done did not include these individuals. The study was held at a site with access to internet and electricity hence the final analysis may not apply to studies done in remote areas.

### Advantages of e-consenting

E-consent was noted to have reduced the burden of documentation and any chance of making errors while participants and study staff were completing the form. Data safety was also enhanced with electronic consenting as consent forms were immediately exported to secure servers thereby reducing the chances of losing the forms and any breach of participant privacy.

### Challenges of e-consenting

Though e-consenting showed to be a reliable tool when conducting research, it also proved that it will be almost impossible to use it in remote areas as it always required a working internet connection to upload the form to allow it to be in a printable pdf format and a printer for printing the participant consent forms as not all areas in Malawi have easy access to electricity and internet. The other challenge we encountered with e-consenting is that some participants were unfamiliar with the tablet user interface such that study personnel needed to be present to troubleshoot where participants experienced difficulties. Since e-consenting is a new concept in Malawi, the ethics review boards did not have digital stamps thus it was initially difficult to digitize consent forms.

### Special considerations of e-consenting

At the time of writing this article, the Malawi Ministry of Health did not have guidelines for electronic informed consenting. As a result, the two IRBs in Malawi (College of Medicine Research Ethics Committee, and National Health Science Research Committee) did not have written guidelines for e-consenting. 

Health Departments or Ministries should have a framework for implementing electronic informed consenting. For example, The US Department of Health and Human Services (HHS), Office for Human Research Protection (OHRP) and Food and Drug Administration (FDA) have jointly developed written guidelines for Institutional Review Boards (IRBs), Investigators and Sponsors for electronic informed consent ^16^. Regulatory entities in the US such as Columbia University IRB and University of Chicago Office of Clinical Research have adopted these regulations and produced guidelines for researchers with interest to implement electronic informed consent ^4, 17^. These guidelines stipulate requirements that must be met for all e-Consenting, including: ensuring all informed consenting elements to be included, use of e-consenting systems that are easy to navigate, stating beforehand how potential volunteers will ask questions to help their understanding, appropriate delegation and training for people obtaining consent, provision of a signed form to the participant either in hard copy or electronically e.g. via email, and ensuring appropriate archiving of consent documents for easy retrieval if needed by the participant, study personnel, sponsors, or study monitors ^4, 16, 17^.

### Feasibility of electronic consenting

Adoption of e-consenting in a clinical or research setting requires some initial financial investment in Information Technology infrastructure for consent platform and secure authentication ^18^. Firstly, electronic devices such as computers, tablets, iPads or cellphones must be purchased. Secondly, supporting software must be installed on the devices. There are several options available; some are open-source ^19^ while others require payment of licensing fees. Examples include: Open Data Kit (ODK), DocuSign, REDCap, and Qualtrics ^4, 17^.

In Malawi, consenting in the clinical and health research domain has been predominantly paper-based. However, with increased digitization and internet access, there is a need to explore if electronic informed consent can supplement paper-based consenting. We set out to pilot electronic informed consent in the first human infection trial in Malawi and to identify gaps, which may need to be addressed to streamline the process in future studies.

## Conclusion

As electronic consent is still a new concept to the Malawian consenting, process there is still a question as to whether e-consenting can be used independently without the paper-based consenting. Electronic consent generally improves the study staff workflow and has been proven a safe and reliable way for performing consenting. These advantages might not be realised in research performed in rural areas and on illiterate individuals, thus there is a need to focus future research in such settings and groups. Nonetheless, electronic consenting has shown to be feasible in Malawian setting and potentially a good strategy for clinical research studies recruiting a large number of participants as it improves overall workflow of studies.

## Data Availability

Figshare: Piloting electronic informed consent: A Pneumococcal Human Infection Study in Blantyre, Malawi. https://doi.org/10.6084/m9.figshare.24321655 ^20^ This project contains the following underlying data: Data file 1 (The attached file contains the following information: Data of participants: Age, Sex, Education level and computer literacy) Data are available under the terms of the Creative Commons Attribution 4.0 International license (CC-BY 4.0).

## Software availability

Data was collected using the Open Data Kit (ODK) application on an android device. To complement to ODK functionality, an additional in-house application was used called ODK lookup updater application, which helped to enforce data validation.
