# A pilot study to explore utility of electronic informed consent in a low- income setting; the case of a Controlled human infection study in Blantyre, Malawi

**DOI:** 10.12688/wellcomeopenres.20770.2

**Published:** 2025-02-05

**Authors:** Clara Ngoliwa, Chikondi Chakwiya, Joel Gondwe, Edna Nsomba, Vitumbiko Nkhoma, Modesta Reuben, Linda Chantunga, Pemphero Liwonde, Edward Mangani, Evaristar Kudowa, Lumbani Makhaza, Neema Toto, Tiferanji Sochera, Tarsizio Chikaonda, Ben Morton, Marc Y.R. Henrion, Dingase Dula, Stephen B. Gordon, Anthony E. Chirwa

**Affiliations:** 1 Malawi-Liverpool-Wellcome Trust Clinical Research Programme, Blantyre, Malawi; 2 Department of Medicine, Queen Elizabeth Central Hospital, Blantyre, Malawi; 3 Department of Clinical Sciences, Liverpool School of Tropical Medicine, Liverpool, UK; 4 Department of Clinical Sciences, Liverpool University Hospitals NHS Foundation Trust, Liverpool, UK

**Keywords:** Electronic informed consent, Open Data Kit, clinical trial, Streptococcus pneumoniae, randomized control trial.

## Abstract

**Background:**

Electronic informed consent can improve accuracy, workflow, and overall patient experience in clinical research but has not been used in Malawi, owing to uncertainty about availability, utility, patient data security and technical support.

**Objectives:**

We aimed to explore the utility of electronic consent (e-consent) in an ongoing human infection study in Blantyre, Malawi.

**Methods:**

The approved paper consent forms were digitized using Open Data Kit (ODK). Following participant information giving by the research staff, healthy literate adult participants with no audio-visual impairments completed a self-administered e-consent and provided an electronic signature. We dual-consented participants by both paper-based and electronic-consenting. Signed e-consent forms were uploaded to a secure study server. Utility of e-consenting was observed by participation rate, user-friendliness, documentation error rate, and staff perception of the overall consenting process.

**Results:**

All 109 participants offered e-consenting accepted participation. E-consenting was user-friendly, had no identifiable documentation errors as compared to 43.1% (n 47/109) error rate with paper-based consenting, and ensured data safety, and unravelled areas for consideration. Challenges with e-consenting included difficult digitization of ethics stamped documents, as well as present but infrequent delays of retrieval of e-consent forms.

**Conclusion:**

E-consenting is feasible, has a utility benefit in a controlled human infection study in Malawi; a low-income country, and can supplement paper-based consenting. Its usefulness can improve the consenting process in research conducted in such settings. Additionally, success of e-consenting requires a careful consideration.

## Plain Language Summary

Informed consent involves educating a patient or participant about the risks, benefits and alternatives of a procedure or clinical research in a format and language that they can understand to achieve voluntary participation. Traditionally paper-based consent has been used but it is not without its limitations thus the need to introduce electronic consent. Electronic consent involves the use of electronic devices to deliver a variety of media including video as well as written words to convey the study details and then secure digital recording to save the informed consent.

We piloted electronic consent in an ongoing human infection study to assess feasibility in Malawi. The approved paper consent forms were digitized and uploaded to an electronic platform. Participants completed dual consent of paper and electronic consent in that order. We then compared issues arising from using both methods.

We found that e-consent was feasible. It proved to be reliable, and minimized documentation errors. We noted, however, that electronic consent could not be done in technologically challenged settings as it required internet connection to help upload forms to secure servers. In addition, researchers are still required to provide printed proof of consent and so current practice would also require a printer to be available. When infrastructure limitations are overcome, e-consent offers improved participant experience and increased data reliability.

## Introduction

Informed consent; an integral part of the ethical conduct of any study, is a process that involves explaining to a patient or study participant the risks, benefits or alternatives of a procedure or clinical research in a format and language that is comprehensible, in order to ensure voluntary participation ^1^. Obtaining an informed consent from a study participant can be verbal or written. The written consent has two main modalities: the traditional paper-based consenting process followed by a participant signature on a paper-consent form or an electronic consenting process followed by an electronic signature in the consent form (also called an e-consent) ^2^.

The traditional paper-based consent; despite its usefulness has some challenges including high documentation errors as high as 50%. These errors for example, include incomplete forms due to missing information, wrong spellings, and lack of legibility in some circumstances ^3^. Additionally, paper-based consent requires physical storage which can be challenging and make remote access limited and physical retrieval of consent documents cumbersome, and usually requires physical presence of both the participant and the consenter ^4– 6^.

E-consenting can be done physically in the presence of the study participant and the consenter (usually a nurse or clinician in the case of clinical research or medical procedure or any other trained staff), or remotely. This method of consenting uses electronic devices such as mobile phone or tablets containing study information for the potential volunteer, who demonstrates an informed consent by providing an electronic signature or thumbprint. E-consenting therefore provides a versatile alternative to the paper-based consent and can be useful in enhancing the consenting process through audio-visual and interactive components for participants requiring such ^2, 5^. Additionally, e-consenting allows several flexibility advantages: can be time saving especially when done remotely hence cutting time spent on travel, can provide appropriate user-friendly interface, forms can be easily accessible remotely, and mitigates the need for physical storage space ^6, 7^.

However, e-consenting is not without limitations. These include cost of developing the initial infrastructure and technology to manage and validate electronic consenting documents ^8^ including the participant information and e-consent forms, need for a reliable Wi-Fi network for consenting data upload, retrieval and updates, security risks including corrupted or deleted forms, and its usability in limited resource settings ^8, 9^.

Although e-consenting has been successfully utilised in different studies and settings, as well as ethical issues explored ^10^, there is no study that has explored the role of e-consenting in a human infection study conducted in a low-and middle income setting (LMIC). We therefore designed a sub-study to pilot the utility and explore considerations for e-consenting in participants enrolled in an ongoing Controlled Human Infection Study (CHIS) in Malawi, a low-income country.

## Methods

### Ethics consultation and approval

In October 2021, through the Data Management Support Unit at Malawi-Liverpool Wellcome Programme in Malawi, we engaged the National Health Sciences Research Committee and proposed to test the usability of e-consenting in a human infection study. NHSRC approved the proposal on 29 October 2021, pilot of e-consenting was conducted from 8 ^th^ February 2022 to 9 ^th^ May 2022.

### Study design

We conducted an observational pilot study nested in a large cohort longitudinal trial investigating the efficacy of Pneumococcal conjugate vaccine using a Human Infection Study ^11, 12^. The main trial, conducted at Queen Elizabeth Central Hospital in Malawi, was approved by NHSRC (protocol 16/07/2519) and was registered in the Pan African Clinical Trials Registry (REF: PACTR202008503507113). The e-consenting pilot aimed at exploring the utility of e-consenting in a Human infection study in Malawi, in a low-income setting.

### Study participants, consenting methods and procedures

E-consenting pilot recruited all willing participants enrolled in the main trial ^12^. Recruited participants were healthy literate adults aged 18–40 years, had no physical disability including audio-visual deficiencies, and had access to a mobile phone. We offered dual consenting (paper-based consenting followed by e-consenting) to all study participants recruited to the pilot.

Consenting for all participants recruited to the main trial ^12^ was physical paper-based consenting. The process had two main steps: firstly, study staff (a nurse or a doctor) providing study information to participants using the participant information leaflet (PIL), and secondly completion of the consent form. The consent form contained two sections: *1)* a ten-item comprehension checklist (participant wrote initials) to ensure participant understanding and agreement to adequacy of study information and voluntariness of study participation, *2)* name and signature the participant and study staff, and finally date and participant identification number (PID). Paper based PIL and consent form were filed and stored in locked cabinets within the research room.

Upon completion of paper-based consenting, all participants were offered e-consenting. This procedure was conducted in the presence of both the participant and the consenter. We allowed a “cooling off” period (approximately 5 minutes) before initiating e-consenting. The process and rationale for e-consenting was explained to study participants *apriori* including a brief demonstration of how to navigate through the e-consent forms. E-consenting adapted all ethics approved documents from the paper-based consenting. These included scanned PIL and consent forms in PDF format and the ten-item comprehension checklist in ODK format. Of note, because of the timing of the e-consenting, we did not require repetition of providing study information- the PIL was not repeated.

Study personnel first linked the electronic consent form to a PID by scanning the PID barcode. The form prompted the participant to write their name. We allowed participants to skip the PIL as this discussion had already been done. Next was the consent form containing the ten-item comprehension list. Instead of initialling, the e-consent form prompted participants to select “I agree” by a single tap at the end of each statement to demonstrate comprehension and agreement to the study information, procedures and voluntariness of participation. Using a stylus, both the participant and the study personnel signed the consent form. We exported the consent form to a secure study data server using an internet connection. We printed a copy of the completed e-consent for the study participant.

We explored several observations in both e-consenting and paper-consenting for comparability. We observed ease of navigating and completing the two consenting methods (e-consent versus paper-based consent), user-friendliness, ease of uploading and retrieving the e-consent including producing print-out copies, as well as reliability of internet connectivity. Additionally, we assessed presence of any documentation errors in both e-consent and paper-based consents based on completeness, accuracy and legibility. We categorised documentation errors into two: writing mistakes (such as inaccurate date, time, spellings, misplaced information, lack of legibility) and missing information (incompleteness). We defined *error rate* in documentation as number of participant forms, of the total consent forms, with any amount of observed errors. We sought feedback on time taken to complete e-consent and paper-based consent forms (and not time taken for the consenting process as e-consent did not require repetition of going through the PIL) from study staff. Through unstructured interviews with the study staff, we explored pros/cons of e-consenting and paper-based consenting.

### Setting up e-consenting in Open Data Kit (ODK)

ODK is an open-source suite of tools designed to help users build information services ^13^. Electronic forms, same as the approved paper-based consenting forms were designed using XLSForm syntax which is an intuitive language. This involved defining the questions or consent statement, response options and other relevant information like signature fields. Once the form was designed, it was converted into XML format using the XLSForm converter tool. The XML files were then uploaded to the ODK server or directly to a mobile device using the mobile app for data collection called ODK collect. Following approval from NHSRC, an identical copy of the NHSRC ethical approval stamp was affixed to the electronic CRF in ODK.

## Results

A total of 109 HIS participants (male 67%, n=73) were recruited in the e-consenting sub-study ( Table 1). All the 109 participants were consented using both paper-based and electronic consenting. All participants were able to navigate through the e-consent and required minimal support. Of note, 63.6% (n= 70) of the participants in this pilot were college students with prior experience of using computers and tablet devices. All recruited participants had prior experience of operating a mobile phone.

**Table 1.** Demographic characteristics of e-consent pilot study participants.

	Male	Female	Total
**n (%)**	73 (67.0%)	36 (33.0%)	109 (100%)
**Age (mean)**	27	27.8	27.3
**Previous computer experience n(%)**	63 (57.3%)	23 (20.9%)	86 (78.2%)
**Education level (Primary) n(%)**	6 (5.5%)	10 (9.1%)	16 (14.5)
**Secondary n(%)**	17 (15.5%)	7 (6.4%)	24 (21.8%)
**Tertiary n(%)**	50 (46.0%)	19 (17.3%)	69 (63.3%)

100% (n=109) e-consent forms were successfully uploaded, retrieved and printed out for copies on the same day of clinic visit. There were no missing forms or reports of breach of data security. Study staff reported experiencing delays on some days (not quantified) largely due to poor internet connectivity but were resolved during the period of the enrolment study visit (this visit took approximately 2 hours), nevertheless, the delays were not as frequent. Though unsolicited, none of the 109 participants who were offered e-consenting refused or raised any concerns with e-consenting.

There was 0% error rate (0 of 109 forms) identified in the e-consent; compared to 43.1% error rate (47/109 forms had errors) in the paper-based consent. Of these, 29.4% (n=32) were writing mistakes by both study personnel and participants, and 13.8% (n=15) were missing information. All study staff involved in the consenting process (a total of 10) consistently reported that completing the e-consent took less time than the paper-based consent. However, this was not objectively measured.

Unstructured interviews with study staff on the pros and cons of e-consenting showed that e-consenting supplemented paper-based consenting and further revealed areas of consideration ^5^ related to staff, participants and the e-consenting infrastructure ( Table 2).

**Table 2.** Pros and cons of electronic consenting.

Category	Pros	Cons	Categorical considerations
**Staff and ** **participant ** **operations**	- Reduced documentation errors - Easy to access consent forms, including remotely	- There was need for dual consenting (paper and electronic) as a hybrid method was adopted	- Accessibility - Impact on study teams - User-friendliness and user-comprehension
**e-consent ** **infrastructure**	- Easy to navigate - Faster to complete	- Need for considerations for participants with audio-visual deficiencies	- Participant familiarity and preference of consenting method - Community acceptability of technology - Complexity of the design of electronic forms - Data security
**Internet facility**	- Enhanced data safety as documents were immediately saved to secure server thus preventing them from loss due to misfiling or misplacement especially if there was more than one study happening at a site	- Difficult upload and retrieval of ethics stamped documents - Could not be done if internet was not available	- Availability and reliability of the internet - infrastructure
**Storage facility**	- Reduced the burden of filing as all forms are stored on data server	- Difficult to access - data when the - internet is unavailable	- Accessibility

## Discussion

This study explored the utility of e-consenting in the first human infection study in Africa investigating the protective effect of PCV13 against experimental pneumococcal carriage ^12^. We showed in this study that e-consenting is feasible in Malawi, a low–income setting, and can be used in participants with different literacy levels. All study participants engaged accepted e-consenting and completed both paper-based and e-consents. This observation; 100% participation and completion of e-consenting has been made elsewhere ^5, 14^.

Furthermore, we observed additional usefulness of e-consenting mainly through massive reduction in documentation errors (zero-identifiable errors), as opposed to the traditional paper-based consenting (43.1% error rate). This observation, much that e-consenting immediately followed paper-based consenting (providing elucidation of the consenting process due to prior-experience/exposure), might have contributed to the observed improvement in errors, is still consistent with lower error rates as low as 1% versus 32% (e-consenting versus paper-based) observed in other studies ^3, 15^.

Additionally, e-consenting enhanced ease of form search and retrieval from the database as well as remote access by the investigators. This advantage has shown some utility benefits in studies obtaining remote consenting, eliminating the cost and time-burden of travel for consenting, as well as in situations where face-to-face physical consenting was deemed unsafe and disadvantageous ^5^. Of note, we did not objectively quantify duration of e-consenting versus paper-based consenting. Nevertheless, study staff reported that e-consenting took less time to complete, and form upload to the server was instant. This could increase efficiency by reducing the burden of physical filing especially in settings where keeping a copy of paper-based consent form is optional. Data safety was also enhanced with electronic consenting as consent forms were immediately exported to secure servers. There were no missing forms and no reported or identified breach of participant privacy.

The strength about this study is that to our knowledge, it is the first study of a large cohort of participants in a low-income setting to explore the usability of e-consenting in a controlled human infection model. Additionally, based on 100% participation rate and successful completion, upload, retrieval and issuance of a physical printed copy of the e-consent form to all study participants approached and recruited to the e-consenting study, not only reaffirms feasibility of e-consenting but also strongly suggests acceptability of the e-consenting process in Malawi. Additionally, at the time of writing this article, the Malawi Ministry of Health did not have guidelines for electronic informed consenting. The pilot therefore provides enthusiasm to further explore participant and staff experiences, considerations for e-consenting including infrastructure set-up, ethical and cultural concerns, as well as participant factors including considerations for the illiterate, and those requiring audio-visual enhancements.

The study had some limitations. Dual consenting was offered serially to all recruited participants hence the study lacked random allocation of the consenting method which would have otherwise enhanced comparability of outcomes of the two consenting methods. We made several observations surrounding e-consenting but did not explore participants’ experience and perceptions of e-consenting. Additionally, the pilot was conducted in an urban setting, at a site with access to internet and electricity hence the observation would not apply to studies done in remote areas. Since e-consenting is a new concept in Malawi, the ethics review boards did not have digital stamps thus it was initially difficult to digitize consent forms.

In conclusion, e-consenting has a utility benefit in research in Malawi, has a role in improving the consenting process and can supplement the traditional paper-based consenting. Success of e-consenting is multifactorial and its adoption requires a consultative and careful consideration.

## Data Availability

Figshare: Piloting electronic informed consent: A Pneumococcal Human Infection Study in Blantyre, Malawi. https://doi.org/10.6084/m9.figshare.24321655 ^16^. This project contains the following underlying data: Data file 1 (The attached file contains the following information: Data of participants: Age, Sex, Education level and computer literacy) Data are available under the terms of the Creative Commons Attribution 4.0 International license (CC-BY 4.0).

## Software availability

Data was collected using the Open Data Kit (ODK) application on an android device. To complement to ODK functionality, an additional in-house application was used called ODK lookup updater application, which helped to enforce data validation.
