# Supplementary material for: A pilot study to explore utility of electronic informed consent in a low- income setting; the case of a Controlled human infection study in Blantyre, Malawi
Source: Wellcome Open Res. 2025 Feb 5;9:233. Originally published 2024 Apr 29. [Version 2] doi: 10.12688/wellcomeopenres.20770.2 (PMC11862358; doi:10.12688/wellcomeopenres.20770.2)
Supplement: Peer review history

# Reviewer report: Reviewer response for version 2

**DOI:** 10.21956/wellcomeopenres.26056.r118699

**Published:** 2025-03-06

**Authors:** Melissa McCradden

**Affiliations:** 1 The University of Adelaide, Adelaide, South Australia, Australia

This paper describes a pilot study primarily of the feasibility of e-consent processes in Malawi. 

 This reviewer has rated the conclusions as not being fully justified by the data. 

 "We showed in this study that e-consenting is feasible in Malawi, a low–income setting, and can be used in participants with different literacy levels."

 I'm not sure that this conclusion is entirely warranted based on the data. This is a high proportion of "college students" (63%) which represent only a subset of the total population. 

 I find Table 1 a bit confusing. It is suggested to use a range for the age (not just the mean) and to indicate whether the education levels are highest completed or otherwise? It's also not clear if the proportion stated is the Yes or No answer to the associated question. For global readers, 'primary', 'secondary', and 'tertiary' should be defined.

 Presumably, the system prompts the user to complete each field and has a set of accepted values? If true, then the low error rate (while a good thing!) is not particularly remarkable. Similarly (and related to the second point, below), if it forces a decision on particular items, how is it ascertained that users are not just clicking through to completion? Might the effect of a study team member being beside them for this process affect the generalizability of the results beyond situations where personnel are in parallel to e-consent?

 A more substantial concern upon reviewing the paper is the validity of the consent itself. Not to say that paper-based consent is perfect (we know it is not), but one might expect an evaluation of the feasibility of e-consent to also assess whether the digitized process offers similar (or better) understanding of the research process, the benefits and risks, the procedures, etc. This would need to involve verification that the participant understood and appreciated the information contained in the e-consent, not just whether they completed the form without missing anything. Perhaps this is a future study for the team, and I think the paper does not over-extend what work is described herein, but it's worth a consideration.

Is the work clearly and accurately presented and does it cite the current literature?

Yes

If applicable, is the statistical analysis and its interpretation appropriate?

Not applicable

Are all the source data underlying the results available to ensure full reproducibility?

Yes

Is the study design appropriate and is the work technically sound?

Yes

Are the conclusions drawn adequately supported by the results?

Partly

Are sufficient details of methods and analysis provided to allow replication by others?

Yes

Reviewer Expertise:

research ethics, pediatric ethics, artificial intelligence, healthcare evaluations

I confirm that I have read this submission and believe that I have an appropriate level of expertise to confirm that it is of an acceptable scientific standard.

# Reviewer report: Reviewer response for version 2

**DOI:** 10.21956/wellcomeopenres.26056.r118171

**Published:** 2025-02-25

**Authors:** Evelyne Kestelyn

**Affiliations:** 1 Oxford University Clinical Research Unit, Ho Chi Minh City, Vietnam

The authors have addressed all my comments satisfactory so I am happy to approve this new version without any further questions or comments.

Is the work clearly and accurately presented and does it cite the current literature?

Yes

If applicable, is the statistical analysis and its interpretation appropriate?

Not applicable

Are all the source data underlying the results available to ensure full reproducibility?

Yes

Is the study design appropriate and is the work technically sound?

Partly

Are the conclusions drawn adequately supported by the results?

No

Are sufficient details of methods and analysis provided to allow replication by others?

Yes

Reviewer Expertise:

Clinical trial conduct, ethics, data management

I confirm that I have read this submission and believe that I have an appropriate level of expertise to confirm that it is of an acceptable scientific standard.

# Reviewer report: Reviewer response for version 2

**DOI:** 10.21956/wellcomeopenres.26056.r118168

**Published:** 2025-02-12

**Authors:** Erisa Sabakaki Mwaka

**Affiliations:** 1 School of Biomedical Sciences, College of Health Sciences, Makerere University, Kampala, Central Region, Uganda

**I have a few more minor comments for the authors:**

**1.  **   The rationale for recruiting only literate people should be included in the methods.

**2. **    How was the unstructured interview data analysed? Content analysis? Thematic analysis? Were these unstructured interviews guided or were respondents just volunteered information? This has to be clear.

**3.  **   A part from College students, who else participated in the study? This information is not included in Table 1.

**Discussion**

**4. **    In paragraph 1 of the discussion, it is stated *“* *We showed in this study that e-consenting is feasible in Malawi, a low–income setting, and can be used in participants with different literacy levels* *”*. I find this statement problematic. The findings of this study cannot be generalized to Malawi because a greater majority of the participants were literate, the sample size was small, and the sampling was non-probability! That statement should be revised.

**5. **    There are two contradicting responses:

 ·       “ *Thank you very much. Both consents were self-administered with supervision by the study staff, we also maintained the same nurses and clinicians during the study. We however did not explore the reason for the errors* *”* *.*

 and, *“* *The errors are due to human mistakes (both study staff and participants) quality control was done at the end of the screening visit* *”*.

 Since it is indicated in the rebuttal that these were human errors, this should be discussed in the paper. There seem to be issues with quality assurance in the trial, particularly with the documentation of informed consent. Even if participants were randomly selected (the sampling method is missing and should be included), that means, there is likelihood that a sizable proportion of the entire cohort has consenting issues. This should not happen in a trial that has good quality assurance protocols. Further, much as this paper is trying to advocate for e-consenting, it would add value to discuss how these errors can be addressed and their occurrence reduced.

**6.** *“* *Additionally, e-consenting enhanced ease of form search and retrieval from the database as well as remote access by the investigators* *”* *.* How was “ease of form search” assessed? Were the nurses and doctors asked to comment on this or this is the authors’ opinion? The discussion should be informed by the study findings.

Is the work clearly and accurately presented and does it cite the current literature?

Partly

If applicable, is the statistical analysis and its interpretation appropriate?

Not applicable

Are all the source data underlying the results available to ensure full reproducibility?

No

Is the study design appropriate and is the work technically sound?

Partly

Are the conclusions drawn adequately supported by the results?

No

Are sufficient details of methods and analysis provided to allow replication by others?

Partly

Reviewer Expertise:

NA

I confirm that I have read this submission and believe that I have an appropriate level of expertise to confirm that it is of an acceptable scientific standard, however I have significant reservations, as outlined above.

# Reviewer report: Reviewer response for version 1

**DOI:** 10.21956/wellcomeopenres.22987.r90963

**Published:** 2024-09-01

**Authors:** Erisa Sabakaki Mwaka

**Affiliations:** 1 School of Biomedical Sciences, College of Health Sciences, Makerere University, Kampala, Central Region, Uganda

1. The study sought to investigate the utility of e-consenting in an ongoing clinical trial. Both paper-based and electronic consenting were done, and the error rates were compared. The participants of the study were mainly college students and community members in Blantyre however, it is not mentioned whether these were trial participants. If so, why were college students enrolled in a clinical trial? The mean age was 27 years, which is higher for average college students. It is difficult to believe that college students can have a mean age of 27 years. This should be explained.

 2. This is an important study that investigated the transition from paper-based to digital consenting, however, the methods, result presentation, and discussion are weak and need major revisions if this paper is to be accepted for Indexing.

 3. The title mentions “pneumococcal human infection study”. yet the manuscript does not address this topic. It might be helpful to revise the title to better reflect the content of the study.

 4. The problem under study is not clearly articulated in the introduction. The objective of the study is also not stated.

 5. The description of "Recruitment to the study of consenting methods" doesn't read well, it should be copy-edited. The description of the methods under the heading "Procedure for comparing consent methods in the Human Infection Study (HIS)" appears to be unclear and somewhat difficult to follow. This section should be recast and copy-edited.

 6. I am not sure whether what is referred to as e-consenting is just signing an electronic form. It is stated on Page 4 "Following completion of paper-based consenting, electronic consenting process was initiated. Study personnel first linked the consent form to a study-assigned participant identification (PID) by scanning the barcodes. The form would then prompt the study personnel to write participant name and their name exactly as written on the paper-based consent form". The elements of consent are well known; the declaration/ signing of the consent document is the final step after someone has understood the consent information and voluntarily agrees to participate in research. Did the e-consenting include all the elements? I.e, disclosure, understanding, and voluntary decision-making, or people were just told to sign an e-form?

 7. It is stated on page 5, that "E-consenting proved to be feasible; it was reliable as it reduced the error rate to 0% (n 0/109) compared to 43.1% (n 47/109) ..." What do the authors mean by "error rate"?

 8. Is it fair to infer that e-consenting is feasible with a small sample size and biased population? The authors should justify this. Reduction in the error rate is not synonymous with feasibility!

 9. Was the paper-based consent self-administered, assisted or both? Might the errors be related to the level of experience of the research assistants?

 10.  Might the errors be due to the lack of a quality control person or data manager?

 11.  How were the pros and cons of e-consenting determined? Was it through interviews? Group discussions? Written short answers? Debriefing meetings? How many staff participated in this activity? Basically, there is not enough details.

 12.  Page 6 states, "The use of electronic consent was noted to have improved consent form accessibility and reduced the burden of the need to print all consent and information documents. It also helped in promoting data safety and reduced the risk of data breach". What do the authors mean by improved consent form accessibility? They have indicated that the consent forms are difficult to access when there is poor internet connectivity, however, in the discussion they mention something to the contrary. This should be elaborated

 13.  The strengths and limitations should be presented at the end of the discussion.

 14. The authors conclude that e-consenting is feasible but, they recruited a biased population of only literate participants, yet it is well documented that in much of sub-Saharan Africa, it is mainly illiterate individuals who participate in research. The authors explored the feasibility of e-consenting however, they seem to have a biased population that affects the credibility of their findings.

 15.  Page 6, "Though e-consenting showed to be a reliable tool when conducting research, it also proved that it will be almost impossible to use it in remote areas as it always required a working internet connection to upload the form to allow it to be in a printable pdf format and a printer for printing the participant consent forms as not all areas in Malawi have easy access to electricity and internet." Statistics on reliability are not presented, how did the authors determine that e-consenting is a reliable tool? How can it be reliable when it is not available in many areas of Malawi that neither have electricity nor internet connectivity?

 16.  The authors have several factual statements and inferences in the discussion and conclusion that are not supported by their findings. They should discuss their findings.

Is the work clearly and accurately presented and does it cite the current literature?

Partly

If applicable, is the statistical analysis and its interpretation appropriate?

Not applicable

Are all the source data underlying the results available to ensure full reproducibility?

No

Is the study design appropriate and is the work technically sound?

Partly

Are the conclusions drawn adequately supported by the results?

No

Are sufficient details of methods and analysis provided to allow replication by others?

Partly

Reviewer Expertise:

NA

I confirm that I have read this submission and believe that I have an appropriate level of expertise to confirm that it is of an acceptable scientific standard, however I have significant reservations, as outlined above.

# Response

**Published:** 2025-01-21

**Authors:** Clara Ngoliwa

**Affiliations:** Malawi-Liverpool-Wellcome Trust Clinical Research Programme, Blantyre, Southern Region, Malawi

The study sought to investigate the utility of e-consenting in an ongoing clinical trial. Both paper-based and electronic consenting were done, and the error rates were compared. The participants of the study were mainly college students and community members in Blantyre however, it is not mentioned whether these were trial participants. If so, why were college students enrolled in a clinical trial? The mean age was 27 years, which is higher for average college students. It is difficult to believe that college students can have a mean age of 27 years. This should be explained.

- Thank you very much. Our study was a sub study under a clinical trial thus all our participants were enrolled in the trial. The mean age of 27 reflects the age for both college students and community members, please refer to the underlying data to see the detailed age for our participants. The main trial under which our study was conducted required that participants be literate. Healthy adults- aged 18 to 40 years including College students, were eligible to participate. We deliberately involved college students to increase likelihood of recruiting literate participants. This has also been the case in our sister CHIMs in Liverpool, UK.

This is an important study that investigated the transition from paper-based to digital consenting, however, the methods, result presentation, and discussion are weak and need major revisions if this paper is to be accepted for Indexing.

- Thank you. This has been agreed and considered. We have extensively revised the paper, re-written all sections and made them stronger.

The title mentions “pneumococcal human infection study”. yet the manuscript does not address this topic. It might be helpful to revise the title to better reflect the content of the study.

- Thank you. We agree and have revised the title.

The problem under study is not clearly articulated in the introduction. The objective of the study is also not stated.

- Thank you. We have revised the introduction, stated the problem, and objectives of the study. The aim has been included in the introduction.

The description of "Recruitment to the study of consenting methods" doesn't read well, it should be copy-edited. The description of the methods under the heading "Procedure for comparing consent methods in the Human Infection Study (HIS)" appears to be unclear and somewhat difficult to follow. This section should be recast and copy-edited.

- Thank you. These sections have been revised.

I am not sure whether what is referred to as e-consenting is just signing an electronic form. It is stated on Page 4 "Following completion of paper-based consenting, electronic consenting process was initiated. Study personnel first linked the consent form to a study-assigned participant identification (PID) by scanning the barcodes. The form would then prompt the study personnel to write participant name and their name exactly as written on the paper-based consent form". The elements of consent are well known; the declaration/ signing of the consent document is the final step after someone has understood the consent information and voluntarily agrees to participate in research. Did the e-consenting include all the elements? i.e, disclosure, understanding, and voluntary decision-making, or people were just told to sign an e-form?

- Thank you very much. Our electronic consent included all elements of informed consent according to ICHGCP standards. E-consenting included participant information sheet, a 10 statement form outlining critical areas of the study, and a signature section for signing. We read the information sheet on physical papers, then asked participants to undergo e-consenting and then sign off. This is clarified more in the paper.

It is stated on page 5, that "E-consenting proved to be feasible; it was reliable as it reduced the error rate to 0% (n 0/109) compared to 43.1% (n 47/109) ..." What do the authors mean by "error rate"?

- Error rate was defined as number of participant forms with any amount of observed errors such as wrong spelling, overwriting, missing participant ID, unfilled sections, wrongly filled sections etc of the total filled consent forms. This definition has been included in the paper.

Is it fair to infer that e-consenting is feasible with a small sample size and biased population? The authors should justify this. Reduction in the error rate is not synonymous with feasibility!

- Thank you. We tested e-consent in a relatively smaller cohort of literate participants. Nevertheless, the numbers tested are not unusual for some observations and interventional studies hence the study still provides useful information to learn from.

Was the paper-based consent self-administered, assisted or both? Might the errors be related to the level of experience of the research assistants?

- Thank you very much. Both consents were self-administered with supervision by the study staff, we also maintained the same nurses and clinicians during the study. We however did not explore the reason for the errors.

Might the errors be due to the lack of a quality control person or data manager?

- The errors are due to human mistakes (both study staff and participants) quality control was done at the end of the screening visit.

How were the pros and cons of e-consenting determined? Was it through interviews? Group discussions? Written short answers? Debriefing meetings? How many staff participated in this activity? Basically, there is not enough details.

- Thank you. This data was determined by both observation and unstructured feedback from study staff (nurses and clinicians) testing e-consent. This is described in the methods section.

Page 6 states, "The use of electronic consent was noted to have improved consent form accessibility and reduced the burden of the need to print all consent and information documents. It also helped in promoting data safety and reduced the risk of data breach". What do the authors mean by improved consent form accessibility? They have indicated that the consent forms are difficult to access when there is poor internet connectivity, however, in the discussion they mention something to the contrary. This should be elaborated

- Thank you. Our study was conducted in a technologically able setting with good internet availability. Instances of not having internet were rare and as such compared to paper based consent accessing the forms was easier than going through large physical folders.

The strengths and limitations should be presented at the end of the discussion.

- Thank you for this, we have revised this in the article.

The authors conclude that e-consenting is feasible but, they recruited a biased population of only literate participants, yet it is well documented that in much of sub-Saharan Africa, it is mainly illiterate individuals who participate in research. The authors explored the feasibility of e-consenting however, they seem to have a biased population that affects the credibility of their findings.

- Thank you. We agree with this observation. However, largely, CHIMs have involved literate participants, both in high-income and low-income settings. The pilot was a sub-study and involved literate participants of an ongoing trial. We agree that future studies should explore utility of e-consent in illiterate participants.

Page 6, "Though e-consenting showed to be a reliable tool when conducting research, it also proved that it will be almost impossible to use it in remote areas as it always required a working internet connection to upload the form to allow it to be in a printable pdf format and a printer for printing the participant consent forms as not all areas in Malawi have easy access to electricity and internet." Statistics on reliability are not presented, how did the authors determine that e-consenting is a reliable tool? How can it be reliable when it is not available in many areas of Malawi that neither have electricity nor internet connectivity?

- Thank you very much, we have rephrased this statement in the article.

The authors have several factual statements and inferences in the discussion and conclusion that are not supported by their findings. They should discuss their findings.

- Thank you. Major revisions to the article have been done.

# Reviewer report: Reviewer response for version 1

**DOI:** 10.21956/wellcomeopenres.22987.r90965

**Published:** 2024-08-12

**Authors:** Dr. Muhammed Afolabi

**Affiliations:** 1 London School of Hygiene and Tropical Medicine, London, UK

The authors reported the findings of their pilot study on the feasibility and usefulness of e-consenting among research participants enrolled in a parent trial in Malawi, a low-income country with an adult literacy level of 68%, according to the 2024 World Bank/UNESCO report ^( 1)^. Given this relatively high literacy level among adult population in Malawi, the authors did NOT support their exclusion of study participants who were not literate in English, with empirical evidence. This selection bias is a major methodological flaw that might have impacted the study findings adversely, as the findings could not be generalised to the adult population in Malawi. Similarly, the inclusion of a large number of students in the study further underscored the skewness and non-generaliseability of the study findings.

 Also, objective comparisons could not be undertaken with the study design adopted by the authors, as the study participants had already undergone the traditional paper consenting before requesting them to repeat the same process using electronic consenting. This introduced a procedural bias into the process. An individually randomised controlled trial would have minimimised this bias and could have generated reliable findings that might have shaped future research or implementation of e-consenting in Malawi.

 The authors did not indicate in their manuscript whether they provided some basic information about e-consenting to the study participants before requesting them to undertake the electronic procedure. The authors' statement that *'Participants were required to simply select I AGREE with a single tap' *suggests an over-simplification of the process and procedures involved in e-consenting. Therefore, this might not have wholly captured the process of e-consenting.

 I was also expecting to see in the results factors that might have shaped the positive results on e-consenting reported by the authors. Could factors such as age, gender, education levels, previous computer experience, ownership of a smartphone, etc, have affected the participant's ability or performance to undertake e-consenting without errors?

Is the work clearly and accurately presented and does it cite the current literature?

Yes

If applicable, is the statistical analysis and its interpretation appropriate?

No

Are all the source data underlying the results available to ensure full reproducibility?

Yes

Is the study design appropriate and is the work technically sound?

No

Are the conclusions drawn adequately supported by the results?

Partly

Are sufficient details of methods and analysis provided to allow replication by others?

Partly

Reviewer Expertise:

Clinical trials, vaccine research, bioethics, global public health

I confirm that I have read this submission and believe that I have an appropriate level of expertise to confirm that it is of an acceptable scientific standard, however I have significant reservations, as outlined above.

## References

[1] 2024 World Bank/UNESCO report.2024; Reference source

# Response

**Published:** 2025-01-21

**Authors:** Clara Ngoliwa

**Affiliations:** Malawi-Liverpool-Wellcome Trust Clinical Research Programme, Blantyre, Southern Region, Malawi

The authors reported the findings of their pilot study on the feasibility and usefulness of e-consenting among research participants enrolled in a parent trial in Malawi, a low-income country with an adult literacy level of 68%, according to the 2024 World Bank/UNESCO report ( 1). Given this relatively high literacy level among adult population in Malawi, the authors did NOT support their exclusion of study participants who were not literate in English, with empirical evidence. This selection bias is a major methodological flaw that might have impacted the study findings adversely, as the findings could not be generalised to the adult population in Malawi. Similarly, the inclusion of a large number of students in the study further underscored the skewness and non-generaliseability of the study findings.

- Thank you very much. Traditionally most CHIMs have been done in HICs and are recently expanding into low and middle income countries. One ethical concern for conducting CHIMs in LMICs is the challenge of obtaining voluntary informed consent especially in participants who are not literate, who may thus be considered as vulnerable (Informed consent for Controlled Human Infection Studies in low- and middle  income countries: Ethical challenges ad proposed solutions (doi: 10.1111/bioe.12795. Epub 2020 Aug 10) The transferred protocols included only the literate. The pilot was a sub-study, the main trial under which the pilot was conducted was the first CHIM in Malawi and it excluded illiterate participants. We agree this method excludes a population which would have otherwise been included. Future CHIMs should conduct prior community consultation on inclusion of illiterate participants. We Agree with the reviewer and we have clarified in paper that this was a sub-study of an ongoing trial that involved only literate participants

Also, objective comparisons could not be undertaken with the study design adopted by the authors, as the study participants had already undergone the traditional paper consenting before requesting them to repeat the same process using electronic consenting. This introduced a procedural bias into the process. An individually randomised controlled trial would have minimised this bias and could have generated reliable findings that might have shaped future research or implementation of e-consenting in Malawi. 

- Thank you. We agree that prior paper based consenting might have improved observations seen in e-consenting. This has been clarified in the findings of the paper.

The authors did not indicate in their manuscript whether they provided some basic information about e-consenting to the study participants before requesting them to undertake the electronic procedure. The authors' statement that 'Participants were required to simply select I AGREE with a single tap' suggests an over-simplification of the process and procedures involved in e-consenting. Therefore, this might not have wholly captured the process of e-consenting.

- Information was given verbally. The select option was to make the process easier and faster but participants had to go through each step/ question prior to selecting I AGREE. This has been revised in the paper.

I was also expecting to see in the results factors that might have shaped the positive results on e-consenting reported by the authors. Could factors such as age, gender, education levels, previous computer experience, ownership of a smartphone, etc, have affected the participant's ability or performance to undertake e-consenting without errors?

- Thank you very much. We agree but unfortunately we did not explore these factors.

# Reviewer report: Reviewer response for version 1

**DOI:** 10.21956/wellcomeopenres.22987.r87017

**Published:** 2024-08-07

**Authors:** Evelyne Kestelyn

**Affiliations:** 1 Oxford University Clinical Research Unit, Ho Chi Minh City, Vietnam

The potential of using electronic consent in LMIC research settings is an exciting development and I read this article with great interest. I would propose a few changes to make this publication's outputs clearer and stronger.

Introduction.

- (Para 1) Educating a patient (in my view) sounds a bit condescending and other words like explaining, detailing might be considered.
- (Para 2) No mention is made about limitations from the patient's side, it only seems to focus on the research centre staff and storage.
- (Para 4) No mention is made about the considerable challenges of keeping digital data secure and protected once it's collected. Given this is all non- anonymised data, keeping in line with current applicable guidelines and maintaining long term data security are challenging.

Methods.

- (Ethics) I would like a bit more clarity about the 'proposal' that was approved. Was this submitted as a separate study within a trial? The study design is categorised as a cross-sectional observational study so this should get separate ethics approval from the clinical trial. This might have been obtained but is not clear from the text.

Results.

 Your result section makes statements that I don't feel are supported by the data presented.

- The authors mention e-consenting is feasible but they should note that this is only the case for participants that have no (visual or hearing) impairments. No mention is made about illiterate participants or how e-consenting might be a help in this population.
- The authors mention it was reliable and mention a 0% mistake rate for the e-consenting. In my opinion, the data presented cannot be interpreted as meaning e-consenting is more reliable as the participant had already gone through a (written) consent procedure. There is no mention of how this could have affected the second consent procedure. Repeating the same actions usually leads to a lower error rate.
- (Documentation errors analysis) It might be better to clearly detail all errors so the reader can understand whether the errors were solely related to spelling, overwriting etc and not to more structural errors and understanding. This section can be broadened.

Discussion.

- (Para 1) Again I think the claim that e-consenting had a 0% error rate compared to paper-based consenting cannot be made based on this study design and results.

Previous studies.

- Did your study look at staff satisfaction or participant usability and satisfaction? Claiming e-consenting is feasible without including any data on the patients' experiences is challenging.

Study limitations.

- The authors rightly point out the lack of randomisation of the two different consent methods. For future studies, they might consider changing the order of obtaining the consent in different groups allowing for a more accurate understanding of the errors and challenges of obtaining e-consent (first and paper-based consent second).

Advantages.

- 'Data safety was enhanced'. This is not supported by any data. Challenges with maintaining this type of data (E- consents) in a long term secure way is not easy and data breaches do occur. Does the site have data about how many forms are lost or where privacy breaches have occurred with paper-based consenting?

Challenges.

- The authors mention that this pilot study was conducted to identify gaps that need to be addressed for streamlining e-consent in future studies. I am unsure what these gaps are and if they are reported in this paper.

Conclusions.

- I think the authors cannot draw the conclusion that e-consenting is feasible in a Malawian setting. It has shown to be feasible in a very specific context only and has not taken into account the participants' perspective.

Is the work clearly and accurately presented and does it cite the current literature?

Yes

If applicable, is the statistical analysis and its interpretation appropriate?

Not applicable

Are all the source data underlying the results available to ensure full reproducibility?

Yes

Is the study design appropriate and is the work technically sound?

Partly

Are the conclusions drawn adequately supported by the results?

No

Are sufficient details of methods and analysis provided to allow replication by others?

Yes

Reviewer Expertise:

Clinical trial conduct, ethics, data management

I confirm that I have read this submission and believe that I have an appropriate level of expertise to confirm that it is of an acceptable scientific standard, however I have significant reservations, as outlined above.

# Response

**Published:** 2025-01-22

**Authors:** Clara Ngoliwa

**Affiliations:** Malawi-Liverpool-Wellcome Trust Clinical Research Programme, Blantyre, Southern Region, Malawi

Introduction. (Para 1) Educating a patient (in my view) sounds a bit condescending and other words like explaining, detailing might be considered.

- Thank you very much for this input. This has been revised and the word "educating" has been replaced with "explaining" in the paper.

(Para 2) No mention is made about limitations from the patient's side, it only seems to focus on the research centre staff and storage.

- Thank you. We did not explore limitations regarding Participants as it was an observation study. This has been added as a limitation in the discussion section.

(Para 4) No mention is made about the considerable challenges of keeping digital data secure and protected once it's collected. Given this is all non- anonymised data, keeping in line with current applicable guidelines and maintaining long term data security are challenging.

- Thank you very much we have revised this paragraph to indicate this and other queries have also been clarifiy.

Methods. (Ethics) I would like a bit more clarity about the 'proposal' that was approved. Was this submitted as a separate study within a trial? The study design is categorised as a cross-sectional observational study so this should get separate ethics approval from the clinical trial. This might have been obtained but is not clear from the text.

- Thank you. This was a Sub study and approval to do a pilot was sought in an amendment. The Study is a product of a review meeting with NHSRC. We have clarified this in the paper and have provided a protocol reference number.

Results. The authors mention e-consenting is feasible but they should note that this is only the case for participants that have no (visual or hearing) impairments. No mention is made about illiterate participants or how e-consenting might be a help in this population.

- Thank you for this observation, we have revised and clarified this statement. We propose that this could be translated into other research studies. The result section has been thoroughly revised.

The authors mention it was reliable and mention a 0% mistake rate for the e-consenting. In my opinion, the data presented cannot be interpreted as meaning e-consenting is more reliable as the participant had already gone through a (written) consent procedure. There is no mention of how this could have affected the second consent procedure. Repeating the same actions usually leads to a lower error rate.

- Agreed. We have revised that statement by adding that our study did not identify any errors. This improvement could be a result of prior paper-based consent process. We have also removed the word etc. from the article.

Documentation errors analysis) It might be better to clearly detail all errors so the reader can understand whether the errors were solely related to spelling, overwriting etc and not to more structural errors and understanding. This section can be broadened.

- Thank you. We have revised this section to be more precise. We have also defined documentation errors to clarified this,

Discussion. (Para 1) Again I think the claim that e-consenting had a 0% error rate compared to paper-based consenting cannot be made based on this study design and results.

- We agree with this. Admittedly no errors were identified with electronic consent because of the choice of study design, this could have been improved with participants already being familiar with the paper-based consent. We have revised the wording in the article.

Previous studies. Did your study look at staff satisfaction or participant usability and satisfaction? Claiming e-consenting is feasible without including any data on the patients' experiences is challenging.

- Thank you very much. The pilot did not explore staff or participant satisfaction. However we sought staff feedback on e-consenting.

Study limitations. The authors rightly point out the lack of randomisation of the two different consent methods. For future studies, they might consider changing the order of obtaining the consent in different groups allowing for a more accurate understanding of the errors and challenges of obtaining e-consent (first and paper-based consent second).

- Thank you, excellent observation. We agree. 

Advantages. 'Data safety was enhanced'. This is not supported by any data. Challenges with maintaining this type of data (E- consents) in a long-term secure way is not easy and data breaches do occur. Does the site have data about how many forms are lost or where privacy breaches have occurred with paper-based consenting?

- Thank you so much. The study file is kept in the institutional database. Our study did not have missing E-Consent forms. No data breaches were identified during this study. We have consulted and confirmed that there were no data breache. We have revised the wording of the article.

Challenges. The authors mention that this pilot study was conducted to identify gaps that need to be addressed for streamlining e-consent in future studies. I am unsure what these gaps are and if they are reported in this paper.

- The paper demonstrates feasibility of e-consent in Human Infection Studies. The study set up had required facilities to support use of an e- consent. The pilot serves as a reference to studies requiring to set up and demonstrate usability by participants. We have Rephrased the sentence from “identify gaps” to “propose considerations.

Conclusions. I think the authors cannot draw the conclusion that e-consenting is feasible in a Malawian setting. It has shown to be feasible in a very specific context only and has not taken into account the participants' perspective.

- Agreed! We suggest studies considering e- consent should have a similar set up and explore participant experiences in detail. We have Revised the wording. And have included statements such as “usefulness, unique advantages, and enthusiasm for utility is studies recruiting large cohorts of participants.

# Reviewer report: Reviewer response for version 1

**DOI:** 10.21956/wellcomeopenres.22987.r82476

**Published:** 2024-06-08

**Authors:** Dr. Francis Masiye

**Affiliations:** 1 Malawi University of Science and Technology, Limbe, Southern Region, Malawi

This paper presents exciting results of a study piloted by e-consent/digital consent. It is well-written and presented. However, the following issues need to be addressed by the authors:

 1. Please provide the NHSRC reference number for the study in the methods section under "Ethics".

 2. There are discrepancies in how potential participants provided consent during their recruitment into the study; under the section on "Recruitment to the study of consenting methods", it is stated that verbal consent was obtained from participants, and yet under the section on "Procedure for comparing consent methods in the HIS", it states that both participants and study staff signed the consent form respectively. Please confirm whether verbal consent or written consent was obtained from participants. If verbal consent was obtained from potential participants, please justify why verbal consent was sought. 

 3. Under the section on "Procedure for comparing consent methods in the HIS", it is stated that participants were quizzed with 10 questions to assess understanding, please explain how the data collected from the 10 questions was analyzed.

 4. Under the section on "Setting up e-consenting in ODK", it is explained that data was collected using the ODK application, please explain how data collected via the ODK platform was analyzed and how the results were arrived at. 

 5. Please clarify why none of the authors declares any conflict of competing interests or lack of it.

Is the work clearly and accurately presented and does it cite the current literature?

Yes

If applicable, is the statistical analysis and its interpretation appropriate?

Partly

Are all the source data underlying the results available to ensure full reproducibility?

No source data required

Is the study design appropriate and is the work technically sound?

Yes

Are the conclusions drawn adequately supported by the results?

Yes

Are sufficient details of methods and analysis provided to allow replication by others?

Partly

Reviewer Expertise:

My areas of research interest are informed consent, post-trial access, fair resource allocation, clinical trial monitoring, and regulatory aspects of clinical research.

I confirm that I have read this submission and believe that I have an appropriate level of expertise to confirm that it is of an acceptable scientific standard, however I have significant reservations, as outlined above.

# Response

**Published:** 2025-01-21

**Authors:** Clara Ngoliwa

**Affiliations:** Malawi-Liverpool-Wellcome Trust Clinical Research Programme, Blantyre, Southern Region, Malawi

This paper presents exciting results of a study piloted by e-consent/digital consent. It is well-written and presented. However, the following issues need to be addressed by the authors: 1. Please provide the NHSRC reference number for the study in the methods section under "Ethics"

- Thank you very much. The NHSRC reference number has been added to the article.

There are discrepancies in how potential participants provided consent during their recruitment into the study; under the section on "Recruitment to the study of consenting methods", it is stated that verbal consent was obtained from participants, and yet under the section on "Procedure for comparing consent methods in the HIS", it states that both participants and study staff signed the consent form respectively. Please confirm whether verbal consent or written consent was obtained from participants. If verbal consent was obtained from potential participants, please justify why verbal consent was sought.

- Thank you. We agree that this indeed is a discrepancy we have revised the sentence under “Recruitment to the study of consenting methods” to read “Participants underwent dual consenting (paper-based and electronic consenting in that order)." to clarify how the consenting processes went.

Under the section on "Procedure for comparing consent methods in the HIS", it is stated that participants were quizzed with 10 questions to assess understanding, please explain how the data collected from the 10 questions was analysed. 

- Thank you. The quiz was in relation to the CHIM study as stated that this was a sub study, the quiz was marked in real time to assess participant comprehension. Pass mark was at least 80%. This data was not analysed.

Under the section on "Setting up e-consenting in ODK", it is explained that data was collected using the ODK application, please explain how data collected via the ODK platform was analyzed and how the results were arrived at.

- Thank you very much. This data was not analysed. The electronic consent form included all components of the physical paper- based consent. We checked for any missing consents, incomplete consents and documentation errors. These have been defined and clarified in the new version of the revised manuscript.

Please clarify why none of the authors declares any conflict of competing interests or lack of it.

- Thank you very much. It was a pilot to inform feasibility of e-consenting in an LMIC setting. This was a sub study conducted by same investigators. The pilot was not designed for any commercialisation.
